# Social Support and Longevity: Meta-Analysis-Based Evidence and Psychobiological Mechanisms

**DOI:** 10.3389/fpsyg.2021.717164

**Published:** 2021-09-13

**Authors:** Jaime Vila

**Affiliations:** Human Psychophysiology and Health Laboratory, Mind, Brain, and Behavior Research Center (CIMCYC), University of Granada, Granada, Spain

**Keywords:** social support, social relationships, health, longevity, meta-analysis, stress, stress-buffering

## Abstract

Over the past 60 years, evidence has accumulated on the fundamental role of supportive social relationships in individual health and longevity. This paper first summarizes the results of 23 meta-analyses published between 1994 and 2021, which include 1,187 longitudinal and cross-sectional studies with more than 1,458 million participants. The effect sizes reported in these meta-analyses are highly consistent with regard to the predicted link between social support and reduced disease and mortality; the meta-analyses also highlight various theoretical and methodological issues concerning the multi-dimensionality of the social support concept and its measurements, and the need to control potential confounding and moderator variables. This is followed by an analysis of the experimental evidence from laboratory studies on psychobiological mechanisms that may explain the effect of social support on health and longevity. The stress-buffering hypothesis is examined and extended to incorporate recent findings on the inhibitory effect of social support figures (e.g., the face of loved ones) on fear learning and defensive reactions alongside evidence on the effect of social support on brain networks that down-regulate the autonomic nervous system, HPA axis, and immune system. Finally, the paper discusses the findings in the context of three emerging research areas that are helping to advance and consolidate the relevance of social factors for human health and longevity: (a) convergent evidence on the effects of social support and adversity in other social mammals, (b) longitudinal studies on the impact of social support and adversity across each stage of the human lifespan, and (c) studies that extend the social support framework from individual to community and societal levels, drawing implications for large-scale intervention policies to promote the culture of social support.

## Introduction: Historical Background

Evolutionary biologists did not anticipate the continuing rise in life expectancy in high-income countries (Kirkwood, 2017). Assuming that the aging process is a non-malleable biological phenomenon, they expected that a reduction in early- and mid-life-mortality to minimal levels, thanks to advances in preventive and therapeutic medicine, would simply result in a larger number of older people dying at the same ages as in previous generations. However, death rates at advanced ages continue to fall in these countries, where the elderly are living longer and generally enjoying better health (Kirkwood, 2017). This surprising phenomenon is attributed to non-genetic factors that affect the aging and longevity of individuals, including environmental contamination, socioeconomic status, smoking, alcohol consumption, body weight, physical activity, and social support (Rizzuto and Fratiglioni, 2014; Stringhini et al., 2017; Chowdhury et al., 2018). Among these, social support-related factors have recently attracted considerable attention across numerous disciplines, including biology, epidemiology, medicine, paleo-anthropology, demography, sociology, and psychology (Snyder-Mackler et al., 2020).

The survival of humans depends on their effective social functioning (Guerra et al., 2012). Caregiving and attachment are key elements of parental love that are essential not only for survival during infancy and childhood but also for physical and psychological well-being throughout life (Taylor, 2010). The relevance of social bonds for health and longevity was first documented over a century ago by the French sociologist Emile Durkheim in *Suicide* (Durkheim, 1897). He examined the different rates of suicide in Europe and found them to be more prevalent among people with fewer social ties, concluding that a lack of social connections (low social integration) was responsible for the higher suicide rates.

The literature on social support and its influence on physical and mental health can be traced back to the publication by Maslow (1943) of his theory of human needs and motivations and to the writings of Bowlby (1969) on his theory of attachment. Maslow postulated a hierarchy of five classes of needs that people are motivated to satisfy for their healthy functioning. These range from the most basic (e.g., food and drink) to the most complex requirements (self-actualization and full potential achievement). Maslow placed social needs (social relationships, love, and friendship) at the middle level of this hierarchy. Bowlby developed his attachment theory, influenced by the work of the ethologist Lorenz (1935), to explain early social development through the formation by children of close relationships with familial caregivers. Conceived as an innate biological system, attachment protects individuals from danger by establishing emotional security through contact and reassurance with an attachment figure, who functions as a safety signal.

However, the idea that social and psychological factors can protect from physical disease and mortality did not fit well with the predominant medical model of the time, based on Cartesian mind-body separation (Uchino, 2004). By the middle of the twentieth century, three new scientific societies helped to promote a shift in the dominant biological paradigm: the American Psychosomatic Society (1942), the Society for Psychophysiological Research (1960), and the Society of Behavioral Medicine (1978). These associations endorsed the “biopsychosocial model” of disease and health, which assumes that social, psychological, and biological factors operate interactively across the life-span to preserve human health (Engel, 1977). Being interdisciplinary in nature, these societies welcomed the idea that social relationships can influence health and longevity and fostered research on the neurophysiological mechanisms that might underlie this influence.

In 1976, two seminal reviews were published on the concept of social support and its effects on health and mortality (Cassel, 1976; Cobb, 1976). The epidemiologist John Cassel centered his review on social support as an example of a social environmental factor, focusing on its capacity to change human susceptibility to disease agents. Social support is defined in terms of the presence of other members of the same species, with the strongest support being provided *by the primary groups of greatest importance to the individual*. According to Cassel, this type of social support acts as a protective factor that buffers the individual from the physiological or psychological consequences of exposure to stressful situations. For his part, the psychiatrist Sydney Cobb, centered his review on the concept of social support, which he defined as information that leads subjects to believe: (a) they are cared for and loved, (b) they are esteemed and valued, and/or (c) they belong to a network of communication and mutual obligation. Both Cobb and Cassel considered that social support protects people by buffering the health consequences of life stresses.

Three years later, in 1979, the epidemiologists Berkman and Syme (1979) published one of the most influential and cited longitudinal studies on social relationships and mortality, the Alameda County study. They surveyed a random sample of 6,928 adults from Alameda County in California with a subsequent 9-year follow-up. They measured all-cause mortality and a social network index made up of four sources of social contact: (1) marriage, (2) close friends and relatives, (3) religion, and (4) informal and formal group/associations. The findings showed that those who had more social and community ties at the outset were less likely to die during the follow-up than were those with fewer social contacts. The link between social ties and mortality was independent of initial health status and health practices, including smoking, alcohol consumption, obesity, and physical activity.

Over the next two decades, there was an explosion of interest in the topic of social support and health, with an exponential increase in the number of scientific articles and academic works. Between 1980 and 2000, no fewer than 13 academic books were published in English on social support and health (Gottlieb, 1981, 1983; Whittaker and Garbarino, 1983; Cohen and Syme, 1985; Litwak, 1985; Sarason and Sarason, 1985; Sauer and Coward, 1985; Lin et al., 1986; Vaux, 1988; Sarason et al., 1990; Shumaker and Czajkowski, 1994; Cohen et al., 2000). It soon became evident that it was necessary to clarify concepts and methods and construct a coherent theory of social support to guide future research and applications. The hundreds of new empirical studies claiming evidence of an association between social support and health markedly differed not only in their definition of social support and its measurement but also in their research strategy and in the strength and direction of reported evidence. Terms such as social support, social relationships, social ties, social connection, social integration, social capital, social networks, were all used interchangeably with little critical examination. The multiple interpretations of the same concept of support led to a variety of typologies and classifications: instrumental, emotional, informational, financial, tangible, perceived, received, objective, and subjective, among others. The studies also differed in research design, ranging from ecological to case-control, cross-sectional, retrospective, longitudinal, and randomized controlled studies. The main problem with the human studies was the causal interpretation of the results, which was always in favor of the social causation hypothesis, i.e., differences in social support were the cause of the differences in health. The correlational nature of most of these studies meant that they could not rule out the reverse causation hypothesis, i.e., differences in health may be responsible for differences in social support.

However, by the final decade of the twentieth century, researchers began to agree on the main conceptual and methodological frameworks in which studies on social support and health should be conducted and evaluated. First, studies were classified according to two general ways of measuring social support: structural measures and functional measures. Structural measures refer to the characteristics of the social network around the individual. These include marital status, number of social relationships, frequency of contacts, or membership of community groups. Terms such as social ties, social connections, isolation, social integration, and social networks would fit the category of structural support. Functional measures refer to the characteristics of the support provided by social networks, including emotional, instrumental, informational, and/or financial support. Terms such as perceived support, received support, tangible support, and objective and subjective support would fall into the category of functional support. Second, evidence from studies should not be evaluated individually. Proper evaluation of the scientific evidence involves the application of quantitative analytic methods to a large number of independent studies, preferably in meta-analyses. Finally, advances in our understanding of the beneficial effects of social support on health and longevity require experimental evidence on the neurophysiological mechanisms involved in the association.

The present paper was designed in accordance with these three conceptual and methodological frameworks. It first offers an initial summary of evidence on the association of functional and structural measures of social support with individual health and longevity, drawn from the results of 23 meta-analyses selected from articles published in the English language between 1994 and 2021. Next, it examines experimental evidence on the neurophysiological mechanisms that may explain this association. Finally, it discusses the findings in the context of three emerging research areas that consolidate and expand the role of social support in health and longevity by incorporating new research perspectives (evolutionary, lifespan, and systemic).

## Human Social Support as Predictor of Individual Health and Longevity

### Evidence From Published Meta-Analyses

Two electronic databases (Scopus and Web of Science) were searched for meta-analysis studies using the following combination of terms: (social support or social engagement or social isolation or social relationship or social network or marital status) and (longevity or mortality or death or disease or health). The electronic searches were restricted to studies published in English with adolescent or adult human participants. They had to include at least one measure of social support as independent variable and at least one outcome measure of longevity/mortality/health/disease as dependent variable. Additional complementary search strategies were used by checking cross-references between the meta-analyses and relevant systematic reviews.

A total of 23 meta-analyses published between 1994 and 2021 complied with the inclusion criteria. They were checked to ensure that they followed the PRISMA protocol and that the final list of primary studies across the 23 meta-analyses did not include duplicate studies or participants. The 23 meta-analyses covered 1,187 non-duplicate primary studies with more than 1,478 million participants. Tables 1–3 summarize the results of the 23 meta-analyses, reporting authors and year of publication, number of published/unpublished primary studies, total participants, design, social support measures, outcome measures, heterogeneity test, publication bias test, effect size measure, and effect size results. The meta-analyses are distributed in the Tables according to type of effect size (correlation vs. risk ratio, odds ratio, or hazard ratio) and outcome measure (health/disease vs. longevity/mortality).

**Table 1:** Summary of Meta-analyses on social support and health/disease using correlation as effect size measure.

**Meta-analysis**	**Published/Unpublished studies**	**Participants + countries-continents**	**Design**	**Social support measures**	**Outcome measures**	**Moderators/Confounders**	**Heterogeneity/Publication bias**	**Effect size measure**	**Summary effect size [95% C.I.]**
Smith et al. (1994)	60/7	23,491 5 countries 3 continents	Longitudinal + cross-sectional	Quantitative Qualitative Functional	Health: Physical health (+) Psychological health (+) Stress (+)	Age, gender Design Study quality	No/Yes	Correlation	0.15 [0.08 0.22]^*^ Physical: From 0.005 to 0.22 Psychological: From 0.095 to 0.158 Stress: From 0.092 to 0.182
Wang et al. (2003)	37/145 (42 with clinical variables)	Over 4,072 Taiwan	Cross-sectional	Combined: General index of social support	Health/Disease: Clinical variables: Health status (+) Physical symptoms (–) Psychological symptoms (–) Depression (–)	NR	Yes/No	Correlation	Health status: 0.25 [0.21 0.29] Physical symptoms: −0.63 [−0.68 −0.59] Psychological symptoms: −0.25 [−0.29 −0.21] Depression: −0.63 [−0.68 −0.58]
Harandi et al. (2017)	51/13	24,002 Iran	Cross-sectional	Combined: General index of social support	Mental health: General health questionnaire (+)	Gender Target population Sampling method Measure instrument	Yes/Yes	Correlation	0.33 [0.283 0.360] Social support questionnaires: From 0.076 to 0.481 Health questionnaires: From 0.306 to 0.52
Guilaran et al. (2018)	24	23,520 7 countries 4 continents	Longitudinal + cross-sectional	Functional: Perceived Received Lack of support Negative support Structural: Social integration	Health/Disease: In disaster responders Positive psychological outcome (+) Negative psychological outcome (–)	Age Gender Health status Measures time lag Type of support Type of responder	Yes/Yes	Correlation	0.19 [NR] Negative psychological outcome for perceived support: −0.20 [−0.25 −0.14] Positive psychological outcome for Perceived support: 0.41 [0.33 0.49]
Schiller et al. (2021)	21	2,273 Western countries Korea Thailand India	Longitudinal + cross-sectional	Functional: Perceived Received	Disease: Depression in family caregivers of children with Autism Spectrum Disorder (ASD) (–)	Type of support Source of support (partner, ASD child, siblings, family, friends, professionals)	Yes/Yes	Correlation	−0.26 [−0.35 −0.18] Perceived: −0.30 [−0.40 −0.18] Received: −0.19 [−0.40 0.04] NS Partner: −0.37 [−0.42 −0.31], ASD child: −0.21 [−0.29 −0.11] Family: −0.27 [−0.41 −0.11], Profess: −0.18 [−0.32 −0.04]
Zalta et al. (2021)	176	88,595 5 continents	Longitudinal + cross-sectional	Functional: Perceived Enacted Negative social reactions Structural: Structural social support	Disease: PTSD symptom severity (–)	Age, Gender Type of support Type of trauma Support provider Various methodological variables	Yes/Yes	Correlation	Cross-sectional studies: −0.27 [−0.30 −0.24] Longitudinal studies: −0.25 [−0.28 −0.21]

*NR, not reported; PTSD, post-traumatic stress disorder; ASD, autism spectrum disorder;*

* *Estimated average [and 95% C.I.] from the weighted effect sizes of the three social support measures*.

**Table 2:** Summary of Meta-analyses on social support and mortality using risk, odds, and hazard ratios as effect size measure.

**Meta-analysis**	**Published/Unpublished studies**	**Participants + countries-continents**	**Design**	**Social support measures**	**Outcome measures**	**Moderators/Confounders**	**Heterogeneity/Publication bias**	**Effect size measure**	**Summary effect size [95% C.I.]**
Pinquart and Duberstein (2010)	87	10,795,137 4 continents	Longitudinal	Functional: Perceived Structural: Network size, marital status	Mortality: Cancer patients	Age, gender, SES Comorbidity, smoking, alcohol Cancer site, cancer stages Time of measures, length of study	Yes/Yes	Risk ratio	Perceived support: 1.22 [1.12 1.33]^*^ Network size: 1.25 [1.14 1.39]^*^ Marital status: 1.15 [1.11 1.19]^*^
Holt-Lunstad et al. (2010)	148	308,849 4 continents	Longitudinal + cross-sectional	Functional: received, perceived, loneliness Structural: marital status, social network, integration, living alone Isolation, complex measure Combined	Mortality: All causes	Age, gender, health status Type of support, cause of mortality Length follow-up, country	Yes/Yes	Odds ratio	1.50 [1.42 1.59] Functional: Odds ratio range: 1.22–1.45 Structural: Odds ratio range: 1.19–1.91 Combined: 1.47 [1.34 1.60]
Barth et al. (2010)	20	34,292 5 countries 2 continents	Longitudinal	Functional: Perceived Structural: Living alone	Mortality: Coronary Heart Disease (CHD) + all causes	Age, SES, smoking Metabolic factors Myocardial function factor	Yes/Yes	Risk ratio or hazard ratio	Functional: 1.59 [1.21 2.08] CHD + all causes Structural: 1.41 [1.17 1.70] all causes Structural: 1.56 [0.94 2.58] NS CHD
Sbarra et al. (2011)	32	6.5 million 3 continents	Longitudinal	Structural: Divorced-separated	Mortality: All causes	Age, gender Length follow-up Number of covariates Country	Yes/Yes	Risk ratio or hazard ratio	1.23 [1.17 1.30] Men: 1.31 [1.23 1.39], Women: 1.18 [1.10 1.27] Age: Younger than 65: 1.31 [1.23 1.39] Older than 65: 1.13 [1.05 1.23]
Roelfs et al. (2011)	94/1	500 million 4 continents	Longitudinal + cross-sectional	Structural: Singles (never married)	Mortality: All causes	Age, gender Baseline at start time Sample size Study quality Country	Yes/Yes	Hazard ratio	1.30 [1.24 1.37] Men: 1.32 [1.23 1.41] Women: 1.23 [1.14 1.32] Gender difference diminishes with time Age: Ratio decreasing at older age from 2.28 to 1.22
Roelfs et al. (2012)	120/4	500 million 4 continents	Longitudinal + cross-sectional	Structural: Widowed	Mortality: All causes	Age, gender, health problems Time elapse from widowed Sample size, study quality Country	Yes/Yes	Hazard ratio	1.20 [1.16 1.25] Men: 1.27 [1.19 1.35], Women: 1.15 [1.08 1.22] Age: ratio decreasing at older age from 1.95 to 1.19 Gender difference diminishes with age (faster for men)
Shor et al. (2012)	102/2	600 million 5 continents	Longitudinal + cross-sectional	Structural: Divorced-separated	Mortality: All causes	Age, gender, SES, health status Life style, life stress, design Length follow-up, Recency div-sep Study quality, country	Yes/Yes	Hazard ratio	1.30 [1.23 1.37] Men: 1.37 [1.27 1.49], Women: 1.22 [1.13 1.32] Age: ratio decreasing at older age from 1.55 to 1.22 Gender difference diminishes with age (faster for men)
Shor et al. (2013)	50	100,000 4 continents	Longitudinal + cross-sectional	Functional: Perceived strength of family ties	Mortality: All causes	Age, gender, health status Source of support (family, friends, others) Degree of support, study quality Country	Yes/Yes	Hazard ratio	1.11 [1.05 1.17] Men: 1.09 [1.00 1.19], Women: 1.13 [1.02 1.25] Family: 1.15 [1.04 1.27], Friends: 0.99 [0.84 1.17] NS Others: 1.15 [1.07 1.24]
Shor and Roelfs (2015)	91	400,000 3 continents	Longitudinal + cross-sectional	Structural: Social contact frequency	Mortality: All causes	Age, gender, health status Source of contact Degree of support, study quality	Yes/Yes	Hazard ratio	1.13 [1.09 1.17] Women: 1.14 [1.04 1.25], Men: NS Non-family members: 1.07 [1.01 1.13] Family members: NS
Holt-Lunstad et al. (2015)	70	3,407,134 4 continents	Longitudinal	Structural: Isolation Living alone Functional: loneliness	Mortality: All causes	Age, gender, SES, health status Depression, physical activity, smoking Cause of mortality, year initial data Length follow-up	Yes/Yes	Odds ratio	1.30 [1.16 1.46] Isolation: 1.29 [1.06 1.56] Living alone: 1.32 [1.14 1.53] Loneliness: 1.26 [1.04 1.53] Age:: <65 OR = 1.57, 65–75 OR = 1.25, >75 OR = 1.14

* *Inverted ratio; SES, socio economic status; NS, not significant*.

**Table 3:** Summary of Meta-analyses on social support and health/disease using risk and odds ratio as effect size measure.

**Meta-analysis**	**Published/Unpublished studies**	**Participants + countries-continents**	**Design**	**Social support measures**	**Outcome measures**	**Moderators/Confounders**	**Heterogeneity/Publication Bias**	**Effect size measure**	**Summary effect size [95% C.I.]**
Gilbert et al. (2013)	39 (10 with social support measures)	3,685,137 USA + international	Cross-sectional	Combined: General index of social support	Health: Self-reported health (+)	Level of analysis: (individual, neighborhood, community) Country	Yes/Yes	Odds ratio	1.30 [1.13 150]
Kuiper et al. (2015)	19	24,966 4 continents	Longitudinal	Structural: Frequency of contact Social participation Functional: Loneliness Satisfaction with network	Disease: Incidence of Dementia or Incidence of Alzheimer Disease (AD)	Age, depression, alcohol Baseline cognition Physical activity functional disability, chronic disease Follow-up time, AD vs. dementia	Yes/Yes	Risk ratio	Frequency of contact: 1.57 [1.32 1.85] Social participation: 1.41 [1.13 1.75] Loneliness: 1.58 [1.19 2.09] Satisfaction social network: NS
Valtorta et al. (2016)	19	181,006 4 continents	Longitudinal	Structural: Social isolation Functional: Loneliness	Disease: Incidence of Coronary Heart Disease (CHD) or Stroke	Age, gender, SES Internal validity Small-study effect	Yes/Yes	Risk ratio	CHD: 1.29 [1.04 1.59] Stroke: 1.32 [1.04 1.68]
Penninkilampi et al. (2018)	33	2,370,452 3 continents	Longitudinal + case-control	Combined: Good social engagement Poor social engagement Functional: Loneliness	Disease: Incidence of Dementia	Age, gender, education Depression, physical activity Study quality, follow-up time Country	Yes/Yes	Risk ratio	Good social engagement:: 1.23 [1.14 1.35]^*^ Poor social engagement: 1.41 [1.21 1.65] Loneliness: NS
Heerde and Hemphill (2018)	52	126,939 4 continents	Longitudinal + cross-sectional	Functional: (Actions by others in support of a distressed individual)	Disease: Youth (aged 12–19) Internalizing behaviors Externalizing behaviors Substance use Educational outcome Bullying victimization	Age, gender, source of support Study design, sample size Country	Yes/Yes	Odds ration	Overall: 1.30 [1.20 1.41]^*^ Internalizing: 1.75 [1.47 2.08]^*^ Externalizing: NS Substance use: 1.35 [1.19 1.54]^*^ Educational outcome: 1.42 [1.29 1.57] Bullying: 1.47 [1.32 1.67]^*^ Victimization: 1.82 [1.64 2.0]^*^
Wen et al. (2020)	7	1,248 Asia: China, India, Nepal Africa: South Africa, Kenia South America: Ecuador, Perú	Group comparison: Support Intervention vs. No intervention	Functional: Material Informational Emotional companionship (integrated measure)	Disease: Drug resistant tuberculosis Treatment success (cured + treat completed) Lost to follow-up (treatment interruption)	Type of outcome Type of social support	Yes/Yes	Odds ratio	Integrated measure: Treatment success: 2.58 [1.80 3.69] Reduction of lost to follow-up: 5.88 [1.82 20]^*^ Without material support: NS
Desta et al. (2021)	13 (4 with social support measures)	1,647 Ethiopia	Cross-sectional	Combined: General index of social support	Disease: Post-partum depression	History of depression Marital status satisfaction Partner violence Substance abuse Quality of study Region (within Ethiopia)	Yes/Yes	Odds ratio	6.27 [4.83 8.13]

* *Inverted ratio; NS, not significant; SES, socio economic status; AD, Alzheimer disease; CHD, coronary heart disease*.

Six meta-analyses used correlation as effect size measure, and the remaining 17 used proportion ratios (risk ratio, odds ratio, or hazard ratio). In the six meta-analyses with effect sizes based on correlation (see Table 1), the outcome measures were health/disease variables assessed as continuous data by means of self-report questionnaires or scales. The social support measures were also assessed as continuous data by means of self-report questionnaires or scales.

Tables 2, 3 exhibit the 17 meta-analyses with proportion ratios (risk, odds, and hazard ratios). The outcome measure of the meta-analyses in Table 2 was mortality (all-cause mortality, cancer mortality, or coronary heart disease mortality). The outcome measure of those in Table 3 was a disease (drug-resistant tuberculosis, depression, Alzheimer disease, dementia, and coronary heart disease). Only one meta-analysis in Table 3 used a positive health variable (mental health) as outcome measure. The social support measures in these 17 meta-analyses were dichotomous variables (e.g., married vs. single people) or dichotomized data from continuous variables (e.g., people with high vs. low scores in social support scales).

The 23 meta-analyses displayed in the three tables obtained highly consistent results, with significant effect sizes confirming the association between social support (regardless of the type of social support measure) and individual health/longevity (regardless of the type of outcome measure). However, as also observed in these tables, the strength of the association depends on numerous factors, including type of effect size, type of outcome, type of social support, and type of moderator variable. In this section, the results are discussed in relation to the type of effect size and type of outcome. The impact of the social support measures and moderator variables are discussed in the following two sections.

The six meta-analyses in Table 1 used correlation as effect size measure. The first three meta-analyses (Smith et al., 1994; Wang et al., 2003; Harandi et al., 2017) examined physical and mental health in adult populations, while the other three (Guilaran et al., 2018; Schiller et al., 2021; Zalta et al., 2021) examined psychological outcomes in disaster responders, depression in family caregivers of autistic children, and post-traumatic stress disorder (PTSD) symptom severity, respectively. The overall effect sizes reported in these meta-analyses ranged from 0.15 to 0.41 for health variables (+) and from −0.20 to −0.63 for disease variables (–). Interestingly, the meta-analysis with lowest effect size (0.15) corresponds to the first published meta-analysis (Smith et al., 1994). The authors concluded that this small effect size, based on 60 primary studies, does not support the assumption that there are *strong, consistently positive relationships between social support and health outcome measures*. They stressed the need for further refinement of social support measures in future research. It is also noteworthy that the most recently published meta-analysis also used correlation as effect size measure (Zalta et al., 2021). The latter meta-analysis was based on almost three-fold more primary studies (*n* = 176) across five continents, used functional and structural measures of social support, analyzed multiple moderator variables, and obtained markedly higher effect sizes (−0.27 in cross-sectional studies and −0.25 in longitudinal studies).

The 10 meta-analyses in Table 2 were based on longitudinal studies (although 5 of them also included cross-sectional studies), and they used risk, odds, or hazard ratios as effect size measures and mortality as outcome measure. All-cause mortality was considered by eight of these meta-analyses, all-cause mortality plus coronary heart disease mortality by one, and cancer mortality by the other. The overall effect size reported for all-cause mortality ranged from 1.11 to 1.53, indicating that the incidence of the outcome was between 11 and 53% more likely in people without social support. The smallest overall effect sizes were obtained by the two meta-analyses that used strength of family ties (1.11) and frequency of contacts (1.13) as social support measures (Shor et al., 2013; Shor and Roelfs, 2015). The largest overall effect size (1.53) corresponded to the meta-analysis that used both structural and functional measures of social support (Holt-Lunstad et al., 2010). Meta-analyses that used structural measures related to marital status (single, divorced-separated, widowed, and living alone) yielded effect sizes between 1.20 and 1.32 (Roelfs et al., 2011, 2012; Sbarra et al., 2011; Shor et al., 2012; Holt-Lunstad et al., 2015). However, as indicated in the table, these effect sizes varied widely when moderator variables such as gender or age were taken into account. With regard to specific types of mortality, effect sizes between 1.15 and 1.25 were obtained for the meta-analysis on cancer mortality (Pinquart and Duberstein, 2010) and an effect size of 1.56 for the one on coronary heart disease mortality (Barth et al., 2010).

The seven meta-analyses in Table 3 used the odds ratio or risk ratio as effect size measure and health or disease as outcome measure, describing effect sizes that ranged from 1.23 to 6.27, indicating that the incidence of the outcome was between 23% and over six-fold more likely in people without social support. The two meta-analyses with the largest effect sizes were related to drug-resistant tuberculosis (Wen et al., 2020), reporting an overall effect size of 5.88 for loss to follow-up in patients without vs. with social support, and to *post-partum* depression (Desta et al., 2021), finding an overall effect size of 6.27 for women without vs. with adequate social support. The remaining meta-analyses reported statistically significant but more moderate effect sizes (1.23–1.82) for mental health (Gilbert et al., 2013), coronary heart disease (Valtorta et al., 2016), and dementia or Alzheimer disease (Kuiper et al., 2015; Penninkilampi et al., 2018) in adults and psychological disorders in young people (Heerde and Hemphill, 2018).

Social support emerges in these meta-analyses as a significant predictor of health and longevity regardless of its conceptualization and measurement. However, this evidence is based on correlational data, i.e., the covariation between observed phenomena. Neither cross-sectional nor longitudinal studies allow causality to be inferred when based on this type of data. It may be suggested by longitudinal studies if one phenomenon precedes the other, but only when all variables affecting the covariation are controlled for, and this is never guaranteed in correlational studies. Two problems arise: the presence of third variables that totally or partially explain the observed association (confounding and moderator variables), and the heterogeneity of effect sizes for primary studies, weakening, or extinguishing the strength of a true association. However, both problems can be addressed and evaluated by applying meta-analysis methodology.

### Sub-Group Differences: The Multidimensionality of the Social Support Concept and Its Measurement

Examination of the social support measures listed in the three tables illustrates the conceptual and methodological diversity described in the Introduction. Twenty-six different social support measures are reported, including: quantitative and qualitative social support; received and perceived social support; enacted social support; network social support; material, informational, and emotional social support; social engagement; and social integration. Only two of these meta-analyses used the recommended functional-structural classification (Cohen and Wills, 1985; Uchino, 2004).

The meta-analysis by Holt-Hunstad and colleagues, published in 2010, was the first to use this classification to examine the risk of mortality associated with three functional measures (received support, perceived support, and loneliness), six structural measures (marital status, social networks, living alone, social isolation, social integration, and a complex measure of social integration), and a combination of both types of measure. Among the structural measures, the complex measure of social integration was the most highly predictive of the mortality risk, with an effect size of 1.91, whereas living alone was the least predictive, with an effect size of 1.19. Among the functional measures, perceived social support and loneliness were the most predictive measures, with effect sizes of 1.35 and 1.45, respectively, whereas received social support did not even reach statistical significance.

The relevance of perceived social support as a predictor of health and longevity is confirmed in five additional meta-analyses in Tables 1, 2 on: health risk in disaster responders (Guilaran et al., 2018), depression in caregivers of autistic children (Schiller et al., 2021), PTSD symptom severity (Zalta et al., 2021), cancer mortality (Pinquart and Duberstein, 2010), and coronary heart disease mortality (Barth et al., 2010). Likewise, the relevance of loneliness (i.e., the perception of inadequate social networks and relationships) as a predictor of disease and mortality was confirmed in the second meta-analysis by Holt-Lundstad and colleagues (in Table 2) on all-cause mortality (Holt-Lunstad et al., 2015) and in two meta-analyses (in Table 3) on dementia (Kuiper et al., 2015) and coronary heart disease (Valtorta et al., 2016).

In general, these data show that the measures that best reflect the multidimensionality of the social support concept (complex measures of social integration or combined measures of functional and structural social support) are the most accurate predictors of a reduced risk of disease and mortality.

### Confounding and Moderator Variables

Correlational studies are based on a non-randomized selection of participants. Evidently, people cannot be randomly assigned to groups for social isolation or divorce. In both cross-sectional and longitudinal studies, the non-randomized selection of participants involves the presence of third variables that can make the association spurious. It is well-known that age, socio-economic status, or physical and mental health at the initial evaluation can influence health status and longevity. Age is by far the most important risk factor for many chronic diseases and disabilities (Kirkwood, 2017), and sociologists have described socio-economic gradients in disease and mortality as a function of income since more than 120 years ago (Snyder-Mackler et al., 2020). Reduced physical and mental health can also hinder the formation of new social ties and lead to the loss of existing relationships, including marriage. Age, socio-economic status, and health can act as confounders in the so-called reverse causation or selection model, which offers an alternative explanation for the association observed between social support and health. According to this type of model, it is the decline in health that explains poor social support, not the other way round.

Moderator variables are those that do not challenge the validity of an association but can affect its strength. They can be methodological (e.g., sample size, study design, or follow-up time) or substantive (e.g., gender, source of social support, or geographical location). In meta-analyses, the influence of these variables is evaluated and controlled by means of sub-group analysis and meta-regression. Overall effect sizes are usually adjusted for the influence of these variables (covariates) or reported separately for specific sub-groups of interest. Tables 1–3 summarize the main moderators controlled in each meta-analysis and the main results of sub-group analyses. In relation to social support and longevity (Table 2), two moderators, besides the aforementioned social support measure, show consistent results: gender and age. The effect of social support, especially in the unmarried, divorced, or widowed, appears to be greater in males than in females and in younger than older individuals. However, there is an interaction between gender and age, observing a decrease in the gender effect at older age, especially in men. Consistent results have not been obtained for the source of social support (e.g., family, friends, or others) or the geographical location of studies, suggesting that the positive effects of social support on health and longevity transcend family, national, and cultural contexts.

The evidence provided by the 23 meta-analyses remains consistent after controlling for confounders and mediators, thereby conferring convergent validity to the predictive role played by supportive social relationships in health and longevity. However, while confounding variables may reverse the causal pathway between social support and longevity, and moderator variables can modify its strength, other variables affecting the causal pathway play a different role acting as mediators between social support and outcome. This is the case of psychobiological mechanisms and, in particular, of the variables involved in dampening the stress response according to the stress-buffering hypothesis.

## Psychobiological Mechanisms Underlying the Association Between Social Support and Longevity

Multiple pathways may link social support with health and longevity. Informational and instrumental support, including financial and material assistance, can help individuals to cope with health problems. Likewise, integration within a supportive social network can prevent health problems by providing positive health role models and reinforcing healthy behaviors (House et al., 1988; Lepore, 1998). These are examples of alternative explanations to the stress-buffering hypothesis. However, neurophysiological and neuroendocrine pathways have been highlighted by researchers ever since the two seminal papers of Cassel (1976) and Cobb (1976). They are involved in mediating activation and inhibition of the stress response in accordance with the so-called the stress-buffering hypothesis.

### The Stress-Buffering Hypothesis

This hypothesis has been defined as the process by which the presence of a conspecific reduces the activity of stress-mediating neurobiological systems (Gunnar and Hostinar, 2015). The concept of stress has been extensively analyzed and investigated since the pioneering studies of Cannon (1929) and Selye (1950) (see *International Encyclopedia of Stress*; Fink, 2007). There is broad consensus across health-related disciplines that three main elements are implicated in stress: (a) a specific type of environmental stimulus, (b) a specific type of biological response, and (c) a specific type of cognitive evaluation of the stimulus and response. From the stimulus perspective, stress requires the presence of a real or interpreted threat to the physical or psychological integrity of an individual (McEwen, 2000). From the response perspective, stress requires the sustained activation of the brain's defense motivational system (Vila et al., 2007). Finally, from the cognitive evaluation perspective, stress requires appraisal of a stimulus as truly threatening and an assessment of defense responses as ineffective to cope with the threat (Lazarus and Folkman, 1984).

The neurobiology underlying the stress response involves a chain of brain activations, starting from the sensory input, proceeding through cortical and subcortical connecting structures (with the amygdala and hypothalamus as critical centers), and ending in autonomic, endocrine, and motor effectors whose function is to protect the organism from the threat (fight-or-flight response). The result is a state of maintained or intermittent activation of physiological and endocrine responses that can, over the long term, compromise the normal functioning of the organs involved and increase the risk of disease and mortality. Two neurobiological subsystems are especially relevant in the above sequence: the hypothalamic-pituitary-adrenocortical (HPA) axis and the sympathetic-adreno-medullar (SAM) axis. Activation of both axes in response to a stressor increases the circulation of glucocorticoids (cortisol) and catecholamines (adrenaline) in the bloodstream to allow energy to be released for the fight-or-flight response, even after the stressor has disappeared.

According to the stress-buffering hypothesis, social support is beneficial for health and longevity because the presence of a bond with social partners attenuates or eliminates the adverse consequences of prolonged HPA and SAM activation. This hypothesis was first formulated by Bovard (1959, 1961, 1962) and was developed in his subsequent publications. Bovard was a neurobiologist interested in the reciprocal inhibition of two zones of the hypothalamus: the posterior zone with catabolic function (via activation of the pituitary- and the sympathetic-adrenal arms of the stress response); and the anterior zone with anabolic function (via parasympathetic activation and growth hormone production). Based on evidence from stimulation and lesion studies in animals and humans, Bovard postulated that social support inhibits the stress response by activating the anterior hypothalamic zone, which inhibits the activity of the posterior zone in a reciprocal manner.

Experimental investigation of the stress-buffering hypothesis in humans has been particularly intensive over the past two decades. The experimental tasks have usually employed laboratory-based stressors, such as public speech, threat of mild electric shock, or exposure to painful stimuli. In children, the tasks may consist in natural stressors such as vaccination injections or exposure to clowns or toys (Gunnar and Hostinar, 2015). Social support manipulation is usually investigated by performance of the task alone or accompanied by an attachment figure or stranger. Two sets of human studies can be differentiated: those focused on the HPA stress response (cortisol reactivity) and those focused on the SAM stress response (cardiovascular reactivity).

The evidence provided by the first set of studies is highly consistent. It was reviewed by Hostinar et al. (2014), Gunnar and Hostinar (2015), and Hostinar and Gunnar (2015), who confirmed the cortisol dampening effect of attachment figures during different developmental stages. They describe a potent parent-child stress buffering during infancy and childhood that becomes less effective in adolescence, when parental buffering starts to switch to friends, followed by a new and powerful romantic partner buffering effect in adulthood. It is noteworthy that the high consistency of these studies may be in part attributable to employment of the Trier Social Stress Test, a well-established stress paradigm to examine cortisol reactivity (Kirschbaum et al., 1993). In this test, the cortisol response appears to be unaffected by the motor components of the stress task (speech and mental arithmetic) and is significantly reduced when the task is performed in the presence of a supportive partner.

The second set of studies offers less consistent evidence, and mixed results have been obtained for cardiovascular stress reactivity in experimental studies testing the stress-buffering hypothesis (Lepore, 1998; Uchino et al., 2011). The reviews by Lepore and Uchino et al. highlight major problems in relation to: social support manipulation (received vs. perceived and passive vs. active), induced stress levels (high vs. low), and conceptual issues related to the match between the stressor demands and the type of support provided (the stress-matching hypothesis). The inconsistent results may also be explained by another important problem in cardiovascular reactivity studies that is not mentioned in the reviews. Unlike in the case of cortisol, autonomic responses are highly sensitive to the interference of motor responses and effort during performance of the task (Gunnar and Hostinar, 2015). Hence, the key issue may not be whether the support provider is active or passive but whether the supported person (participant) is active or passive, and all experimental tasks used in these studies (speech, mental arithmetic, controversial discussion, video game, or Stroop task) require the participant to be highly active.

### The Inhibitory Role of Social Support Figures in Defense Reactions and Fear Learning

There are two well-established paradigms to examine autonomic reactivity to stress-related stimuli without requiring an active participant: Lang's startle probe paradigm and Pavlov's classical conditioning. The startle probe paradigm, developed by Lang and colleagues (see Lang et al., 1990, Lang, 1995, and Lang and Davis, 2006), uses a passive picture-viewing procedure to examine the capacity to potentiate or inhibit defensive responses (e.g., startle reflex) of positive and negative emotions elicited by pleasant, neutral, and unpleasant pictures selected from the *International Affective Picture System* (IAPS; Bradley and Lang, 2007). The paradigm also includes the recording of a wide set of peripheral and central physiological measures (autonomic, somatic, and brain responses) while the participant passively views the pictures. Taken together, these responses make it possible to trace not only the neurobiological circuits underlying the activation of positive and negative emotions but also the brain circuits involved in the emotional potentiation and inhibition of defense reactions.

In the past decade, various studies have applied the startle probe paradigm to investigate the stress-buffering hypothesis by replacing the IAPS pictures with images of attachment figures (face of romantic partner, father, mother, and/or best friend) and comparing with control pictures (face of unknown people, famous people, and/or mutilated faces from the IAPS). The first three studies used the standard startle probe paradigm to confirm that attachment figures elicit a genuine positive emotional response that is not confounded by familiarity or undifferentiated emotional arousal (Vico et al., 2010; Guerra et al., 2011) and is capable of inhibiting the startle reflex (Guerra et al., 2012). The last two studies (Sánchez-Adam et al., 2013; Vila et al., 2019) used an adaptation of the paradigm to examine the brain structures involved in these responses with functional magnetic resonance imaging (fMRI). The results of the first three studies were highly consistent: the same pattern of peripheral and central physiological responses was elicited by faces of attachment figures (in black and white with no emotional expression) as was elicited by the most pleasant IAPS pictures, i.e., intense startle inhibition accompanied by a brief heart rate acceleration, higher skin conductance, greater zygomaticus muscle activity, and increased event-related potentials (P300 and LPP). The last two studies revealed that attachment faces activate brain areas related to the processing of positive emotions (medial orbitofrontal cortex), empathy and subjective happiness (anterior cingulate), and autobiographical memories and identity recognition (posterior cingulate and precuneus).

Eisenberger et al. (2011) recently investigated the face of an attachment figure as inhibitor of stress-related responses in studies on pain perception and fear learning (Hornstein et al., 2016, 2018; Hornstein and Eisenberger, 2017). Using a passive picture viewing procedure within an fMRI scanner, female participants received painful thermal stimulation of two intensities (moderate and high) at regular intervals while viewing pictures of their romantic partner or of a stranger or object. Participants reported reduced subjective pain when viewing the partner vs. stranger or object, showing increased activity in safety-signal brain areas (ventromedial prefrontal cortex) and reduced activity in pain-related brain areas (dorsal anterior cingulate and anterior insula). In addition, neural activity in the safety-signal area was negatively correlated with neural activity in the pain-related areas and with self-reported pain.

Studies by Eisenberger on social support figures during fear learning used a Pavlovian shock conditioning procedure to condition the skin conductance response to faces of social support figures (“*the two individuals that give you the most social support on a daily basis*”) in comparison to faces of strangers or known people or neutral objects. The results obtained demonstrate that in comparison to faces of strangers and neutral objects, social support faces, either presented alone or paired with control faces, act as safety signals with the following capacities: (a) to inhibit their own fear conditioning (Hornstein et al., 2016), (b) to inhibit the expression of fear toward previously conditioned stimuli (Hornstein et al., 2016), (c) to inhibit the fear conditioning of new stimuli (Hornstein and Eisenberger, 2017), and (d) to enhance the extinction of conditioned fear responses and prevent the return of fear after a fear reinstatement procedure with additional shocks (Hornstein et al., 2018). Based on this evidence, Eisenberger and colleagues suggested that social support figures have become *biologically prepared* safety stimuli, analogous to *biologically prepared* fear stimuli (Seligman, 1971; Öhman, 1986), *because over the course of evolutionary history they have provided individuals with protection, care, and resources, which has ultimately promoted survival* (Hornstein et al., 2016, p. 1,051).

### Neurobiological Pathways Involved in the Influence of Social Support on Health and Disease

As already commented, the neurobiological mechanisms underlying the social support effect on health and disease processes are directly linked to activation and inhibition of the defense motivational system by danger and safety signals. Interestingly, the main sources of danger and safety for humans, and probably for other social mammals, are not physical but social stimuli, i.e., the “other.” In 1986, Arne Öhman described fear of the predator (the beast) and fear of social rejection (the other) as the two main ancestral fears. Likewise, the main safety signals with capacity to inhibit fear and defense reactions are also other people: attachment and loved figures.

The brain structures at the core of the defense motivational system are two subcortical areas within the temporal lobes: the amygdala and the bed nucleus of stria terminalis (part of the so-called extended amygdala). Knowledge of these structures derives from animal and human studies on defense reactions and fear conditioning (LeDoux, 1996; Lang et al., 2000; Lang and Davis, 2006). The amygdala receives danger-related sensory information from cortical structures via its lateral and basolateral nuclei, which project to the central nucleus of the amygdala and from there to the bed nucleus of stria terminalis. These two last structures have similar efferent connections to the hypothalamus and to other brainstem areas that directly control specific defense reactions such as freezing (central gray), the startle response (nucleus reticularis pontis caudalis), or the fight-flight response (lateral and paraventricular nuclei of the hypothalamus). The hypothalamic defense areas are of special interest because they mediate activation of the sympathetic branch of the autonomic nervous system (lateral hypothalamus) and the neuroendocrine system (paraventricular nucleus), which play a key role in sustaining activation of the defense system and stress response. In fact, it is chronic activation of the defense system, also called the “default stress response” (Brosschot et al., 2018; Thayer et al., 2021), which transforms the fight-or-flight response from an adaptive response that allows survival to a maladaptive response that promotes disease and mortality. Three subsystems are involved in this transformation and its potential reversal by social support.

#### The Cardiovascular System and Heart Rate Variability

Prolonged activation of the defense system leads to a cardiovascular and autonomic imbalance in which the sympathetic tone is high and the parasympathetic tone is low, a condition associated with increased morbidity and mortality (Thayer et al., 2010, 2021). Inhibition of the defense system by safety signals is accomplished through structural and functional inhibitory connections between areas of the prefrontal cortex (orbitofrontal cortex and medial prefrontal cortex) and amygdala (Thayer and Lane, 2009). Julian Thayer and coworkers recently reported that the autonomic imbalance produced by prolonged activation of the defense system and the inhibitory control of the prefrontal cortex on the amygdala were linked to the aging process, describing a deterioration in both phenomena (greater sympathetic dominance and lower prefrontal inhibition) with increasing age but only up to around 70–80 years (Zulfiqar et al., 2010; Almeida-Santos et al., 2016). Above this age, there is an increase in parasympathetic dominance, measured using indices of vagally-mediated heart rate variability (high frequency variability associated with respiratory sinus arrhythmia), to levels typical of younger individuals. Consequently, it has been suggested that heart rate variability can be used not only as an index of health but also as an index of biological age and longevity (Zulfiqar et al., 2010; Thayer et al., 2021).

Two influential theories, Porges's polyvagal theory (Porges, 2009) and Thayer's neurovisceral integration theory (Thayer and Lane, 2000), uphold that safe environments promote parasympathetic dominance, leading to increased health and longevity. The polyvagal theory posits that when the environment is perceived as safe there is an increased parasympathetic control by the mammalian myelinated vagus, slowing the heart, inhibiting the fight-or-flight response, dampening activity of the HPA axis, and reducing inflammation by modulation of the immune system. This effect is accompanied by activation of an integrated social engagement system via neural links with the face and head muscles that control eye gaze and facial expression, thereby promoting supportive social connection and communication (Porges, 2009). Thayer's neurovisceral integration theory assumes a reciprocal interconnection between the brain and the heart via a complex neural network that comprises the prefrontal cortex, anterior cingulate, amygdala, hypothalamus, and vagus nerve as key structures. This network integrates cognitive, affective, and autonomic systems in a dynamic model that explains emotion and stress regulation/dysregulation (Thayer and Lane, 2000). In this model, heart rate variability is seen as a marker of stress (low variability) and health (high variability), as supported by neuroimaging studies that have revealed associations between heart rate variability and specific brain regions in the prefrontal cortex and anterior cingulate in response to perceptions of safety and threat (Thayer et al., 2012).

#### The HPA Axis and Oxytocin

Similar brain structures to those involved in regulation of the cardiovascular system participate in regulation of the HPA axis. Activity of this axis originates in the parvocellular neurons of the paraventricular nucleus of the hypothalamus by secreting corticotropin-releasing hormone (CRH), which stimulates production of adrenocorticotropic hormone (ACTH) by the anterior pituitary and its release into the general circulation. The ACTH then stimulates the production and release of glucocorticoids (cortisol) by the adrenal cortex, whose main function is the mobilization of energy to cope with environmental challenges. Brain control of the HPA axis uses the same structures as those involved in cortical and subcortical regulation of the cardiovascular system: orbitofrontal and medial prefrontal cortex, anterior cingulate, amygdala, and bed nucleus of stria terminalis (Hostinar et al., 2014). As in the cardiovascular system, these integrated structures mediate activation and inhibition of the HPA axis in response to the perception of threat and safety, thereby contributing to explain the stress-buffering effect of social support.

Another neuroendocrine system that contributes to the social buffering effect is the oxytocin system. The neuropeptide oxytocin is mainly produced by magnocellular neurons of the paraventricular nucleus of the hypothalamus and is released into the circulation by the posterior pituitary. Oxytocin was first recognized for its role in parturition and lactation (Freund-Mercier et al., 1988), while its release in the brain was later found to be responsible for promoting the formation of mother-offspring bonds (Carter, 1998). More recent research, both in animals and humans, has described a role for oxytocin in the regulation of HPA activity, both in direct response to a stressor and in response to a supportive conspecific (Heinrichs et al., 2003; Crockford et al., 2018). Crockford and colleagues have suggested that the release of oxytocin in response to a stressor may facilitate the activation of social-support-seeking behavior. Indeed, finding social support may reduce the threat for an individual, as when chimpanzees face a predator or an aggressive conspecific. In the absence of a stressor, the social support effect may be mediated by the oxytocin-induced downregulation of the HPA axis. Chimpanzee studies have shown that grooming or food sharing is associated with higher urinary oxytocin and lower urinary glucocorticoids when done with bonded vs. non-bonded partners (Wittig et al., 2016; Samuni et al., 2017). These findings confirm that being in a supportive social environment *per se*, without exposure to a stressor, is a health promotion mechanism.

#### The Immune System and Inflammation

Inflammation is the defensive response of the immune system to protect the organism from injury and infection. Eisenberger et al. (2017) recently reviewed evidence that proinflammatory cytokines, immune system markers, have a profound influence on the brain, altering social behavior in opposite directions: (a) increasing sensitivity to negative social experience (e.g., social exclusion) and (b) increasing sensitivity to positive social experience (e.g., viewing pictures of loved ones). Both influences affect sickness behavior, either by withdrawing people from social contacts that may represent an additional threat to well-being or by bringing them closer to attached individuals who may provide support and care to recover from sickness. Eisenberger and coworkers also reviewed evidence that negative social experience has a strong influence on the immune system by increasing proinflammatory cytokines in various social adverse conditions, including real-world social stressors (e.g., parental separation in early life or bereavement in later life), laboratory stressors (e.g., the Trier Social Stress Task), and social disconnection (e.g., social isolation and loneliness).

The association between low social support and inflammation was confirmed in a recent meta-analysis published by Uchino et al. (2018), based on 41 studies with over 73,000 participants. They found a significant negative correlation effect size (Fisher Zr transformation) of −0.073, indicating that low social support is a significant predictor of inflammation. Three types of social support measure were analyzed: social integration (a complex measure including such aspects of the social network as marriage and volunteer organizations), perceived support, and received support. Although no significant differences were found between the three measures, the largest effect size was for social integration (−0.076), followed by perceived support (−0.054) and then by received support (−0.040), which was not statistically significant. These results are consistent with the findings of the present 23 meta-analyses on the superiority of complex social integration measures and perceived support over received support as predictors of health and longevity. The authors acknowledged that the overall effect size was low and that sample sizes were low for the subgroup, calling for further research. This is a highly relevant issue, given that chronic inflammation associated with low social integration and social support can impact on multiple diseases that represent the leading causes of disability and mortality worldwide, including cardiovascular disease, cancer, diabetes mellitus, chronic kidney disease, non-alcoholic liver disease, and autoimmune and neurodegenerative disorders (Furman et al., 2019).

## Discussion and Conclusions

Highly consistent evidence has accumulated over the past 60 years on the significant association of functional and structural measures of social support with health and longevity. The strength of the association varies widely according to the type of social support measure and the type of health outcome. The strongest association has been observed for structural-type complex social integration measures and functional-type perceived support measures and for outcome measures of specific or all-cause mortality. The strength of this association is equivalent to that documented for other well-documented risk factors such as smoking or obesity.

There has also been highly consistent experimental evidence, especially from the past two decades, on three neurobiological pathways that link social support with health and longevity: the autonomic nervous system, the neuroendocrine system, and the immune system. These systems are all sensitive to environmental social cues that activate or inhibit defensive responses. Threatening social cues activate responses in the three systems to protect the organism by increasing cardiovascular activity, cortisol production, and inflammation. However, if sustained for prolonged periods, these same responses can increase the risk of disease and mortality. Conversely, safety social cues induce the inhibition of defense responses, promoting homeostatic levels and social bond formation through parasympathetic dominance, HPA regulation, and oxytocin production, contributing to a reduction in the risk of disease and mortality.

The strongest evidence on the role of social support as safety cues derives from human experimental studies that tested the stress buffering hypothesis using attachment figures (romantic partner, parents, and friends). The results highlighted the emotional component of social support, principally from family and friends, which is identified as love. Love is embedded in the first and most cited definition of social support proposed in 1976: *Information leading to believe that one is loved and cared, esteemed and value, and part of a social network of mutual obligation* (Cobb, 1976). Social psychologists were the first to study romantic and non-romantic love (Mikulincer and Goodman, 2006), describing three basic components: attachment (connection), care giving-receiving (protection), and attraction (sexual attraction in romantic love and positive affect in non-romantic love). Indeed, the concept of love includes the notions of aid (care giving) and connection (attachment) that are inherent to the concept of social support. Moreover, focusing on the emotional component of social support can help to advance knowledge on the brain mechanisms that mediate the longevity effect (Bartels and Zeki, 2004; Vila et al., 2019). Importantly, it can lead to a reorientation of intervention research toward fostering emotions that strengthen collaboration between individuals and groups.

Nevertheless, research on social support and longevity needs to incorporate recent developments within the field that are helping to expand evidence on the link between social support and health/longevity, to advance knowledge on its underlying neurobiological mechanisms, and to translate this knowledge into the design and implementation of large scale preventive public health interventions that increase the culture of social support. These new developments derive from three different perspectives: the evolutionary, the life span, and the systemic.

### The Evolutionary Perspective: Convergent Evidence From Other Social Mammals

Recent comparative studies between human and non-human social mammals have demonstrated that measures of social support and integration in non-human social mammals are strong predictors of health and survival, as observed in humans, with odds ratios between 1.23 and 1.72 (Snyder-Mackler et al., 2020), highly similar to those obtained in the present review of 23 meta-analyses. This association has been demonstrated in at least four mammalian orders: primates, rodents, ungulates (wild horses), and whales. The bulk of the evidence comes from primate studies, which also provide the strongest backing for the social causation hypothesis and, in particular, for biological processes that explain the stress-buffering effect of close social partners. In male Barbary macaques, for example, the company of bond partners (friends) was found to attenuate the stress response to social (received aggression) and environmental (cold temperature) stressors, as reflected in lower fecal glucocorticoids (Young et al., 2014). Similar findings have been reported in chimpanzees (Crockford et al., 2018). The advantage of non-human animal research on the social determinants of health and survival is the possibility to experimentally control the sources of both social adversity and social support. An additional benefit of findings in primates is their close evolutionary proximity to humans. As extensively documented by the primatologist Frans de Waal in *Mama's last hug* (de Waal, 2019), primates share all social emotions with humans, including love, empathy, gratitude, and a sense of justice, the pillars that sustain supportive social relationships.

### The Lifespan Perspective: The Effect of Social Support From Childhood to Late Adulthood

The developmental approach to the stress buffering hypothesis adopted by Gunnar and Hostinar (2015) represents the first effort to apply the lifespan perspective to social support research, with special emphasis on infancy and childhood. A vast amount of evidence has subsequently accumulated from animal and human studies on the negative and positive health consequences of early life experiences. The magnitude of this effect is illustrated by two studies in animals and humans. In the animal study, the lifespan was around 10 years shorter in yellow baboon females who had experienced early life maternal loss or maternal social isolation than in those who had not (Tung et al., 2016). In the human study, living in a loving and caring family was found to reverse the expected negative effects of the short/short polymorphism in the serotonin transporter gene, which is associated with depression and other psychopathologies (Taylor, 2010). More recent research has gone beyond infancy and childhood, focusing on social support effects from adolescence to young, middle, and late adulthood. Yang et al. (2016) combined data from a set of extensive longitudinal studies and demonstrated that indices of social integration exert a differential impact on biomarkers of inflammation, cardiovascular risk, and obesity according to the lifespan stage. This new approach to understanding how the link between social support and longevity unfolds over the lifespan has practical implications for the design of effective intervention policies adjusted to developmental changes.

### The Systemic Perspective: From the Individual to Society

The systemic approach to social support and longevity, recently defended by Holt-Lunstad et al. (2010, 2015) and Holt-Lunstad (2018), represents a means of broadening the social support field through recognition of its multidisciplinary and multilevel character. In common with all social phenomena, social relationships are embedded in four interrelated dimensions: the individual, the family and close relationships, the community, and society. Application of the systemic perspective to research on social support yields two concrete benefits. First, the multiple causal pathways by which social relationships become a risk or a protective factor can be reorganized into a hierarchy of levels of influence, i.e., the individual (e.g., factors related to biological predispositions), the family and close relationships (e.g., factors related to attachment bond formation and early life experiences), the community (e.g., neighborhood and local environmental factors), and society (e.g., factors related to social and cultural norms). Second, application of this approach can support the implementation of more effective preventive interventions analogous to other well-established public health interventions for risk factors such as smoking or obesity. To date, intervention studies designed to increase social relationships have not yielded convincing results (Hogan et al., 2002; Fakoya et al., 2020), which is likely due, at least in part, to their limitation to the individual or family level.

### Conclusions: Toward a Culture of Global Social Support

Loneliness, the perception of social isolation, is reaching epidemic proportions among the elderly in developed countries and is expected to increase further over the next few decades (Cigna, 2020). Social adversity is also increasing in many underdeveloped countries due to war, social conflict, or poverty, mainly affecting children and younger adults (Pettersson and Öberg, 2020). The key question is whether social support interventions can help to reduce the disease and mortality risk associated with such extreme adverse social conditions. Love is the positive emotion that connects people. Attachment, care giving-receiving, and positive affect always have others as the reference point. The feeling of belonging to a social group or community is based on socio-emotional relationships of love and support. Research on social support intervention may need to explore strategies for expanding and strengthening a global rather than merely local or national sense of belonging to a community (de Rivera and Carson, 2015). Raising awareness that we are all one people and that we are all interdependent and connected worldwide requires work to shift prevailing societal norms and values, which focus so narrowly on individualism and local or national group identities. The need for efforts in this direction is the implicit message conveyed by the three research areas emerging in the social support literature. Finally, the widespread utilization of internet-based social networks is a novel phenomenon that warrants future in-depth research to address their role in providing individuals with positive or negative social support.

## Author Contributions

The author confirms being the sole contributor of this work and has approved it for publication.

## Conflict of Interest

The author declares that the research was conducted in the absence of any commercial or financial relationships that could be construed as a potential conflict of interest.

## Publisher's Note

All claims expressed in this article are solely those of the authors and do not necessarily represent those of their affiliated organizations, or those of the publisher, the editors and the reviewers. Any product that may be evaluated in this article, or claim that may be made by its manufacturer, is not guaranteed or endorsed by the publisher.
